# Gene-Based Association Analysis Suggests Association of *HTR2A* With Antidepressant Treatment Response in Depressed Patients

**DOI:** 10.3389/fphar.2020.559601

**Published:** 2020-12-03

**Authors:** Chung-Feng Kao, Po-Hsiu Kuo, Younger W.-Y. Yu, Albert C. Yang, Eugene Lin, Yu-Li Liu, Shih-Jen Tsai

**Affiliations:** ^1^ Department of Agronomy, College of Agriculture and Natural Resources, National Chung Hsing University, Taichung, Taiwan; ^2^ Advanced Plant Biotechnology Center, National Chung Hsing University, Taichung, Taiwan; ^3^ Institute of Epidemiology and Preventive Medicine, National Taiwan University, Taipei, Taiwan; ^4^ Department of Public Health, National Taiwan University, Taipei, Taiwan; ^5^ Yu’s Psychiatric Clinic, Kaohsiung, Taiwan; ^6^ Division of Interdisciplinary Medicine and Biotechnology, Beth Israel Deaconess Medical Center, Harvard Medical School, Boston, MA, United States; ^7^ Institute of Brain Science, National Yang-Ming University, Taipei, Taiwan; ^8^ Brain Medicine Center, Taoyuan Psychiatric Center, Taoyuan City, Taiwan; ^9^ Department of Biostatistics, University of Washington, Seattle, WA, United States; ^10^ Department of Electrical & Computer Engineering, University of Washington, Seattle, WA, United States; ^11^ Graduate Institute of Biomedical Sciences, China Medical University, Taichung, Taiwan; ^12^ Center for Neuropsychiatric Research, National Health Research Institutes, Zhunan Town, Taiwan; ^13^ Department of Psychiatry, Taipei Veterans General Hospital, Taipei, Taiwan; ^14^ Division of Psychiatry, National Yang‐Ming University, Taipei, Taiwan

**Keywords:** gene-based analysis, major depressive disorders, antidepressant, 5-hydroxytryptamine receptor 2A, single nucleotide polymorphism

## Abstract

The serotonin [5-hydroxytryptamine (5-HT)] system has been implicated in the pathogenesis of major depressive disorder (MDD). Among the 5-HT receptor subtypes, 5-HT2 is one of the major pharmacological therapeutic targets for MDD. There have been inconsistent findings in previous pharmacogenetic studies investigating the antidepressant therapeutic response using one or several 5-HT2A (*HTR2A*) genetic polymorphisms. By using gene-based association analysis, we hope to identify genetic variants of *HTR2A* which are related to MDD susceptibility and its antidepressant therapeutic response. 288 *HTR2A* single nucleotide polymorphisms in MDD susceptibility have been investigated through a case–control (455 MDD patients and 2, 998 healthy controls) study, as well as in antidepressant efficacy (*n* = 455) in our current research. The 21-item Hamilton Rating Scale for Depression was used to evaluate measures of antidepressant therapeutic efficacy. From two MDD groups in the antidepressant therapeutic response, by using gene-based analyses, we have identified 14 polymorphisms as suggestive markers for therapeutic response (13 for remission and 1 for response) in both meta- and mega-analyses. All of these *HTR2A* reported polymorphisms did not reach statistical significance in the case–control association study. This current investigation supported the link between *HTR2A* variants and antidepressant therapeutic response in MDD but not with MDD susceptibility.

## Introduction

One of the common and sometimes fatal mental disorders is major depressive disorder (MDD), which is also a leading cause of disability worldwide (Moussavi et al., 2007). Even with the large number of studies, MDD pathogenesis and the mechanism of drug action remain to be elucidated. Accumulating evidence from various studies such as adoption, family, and twin studies have shown that genetic factors play major roles in MDD development (Flint and Kendler, 2014; Shadrina et al., 2018). For example, a heritability of 31–42% is suggested by twin and adoption studies, with the heritability level possibly higher for reliably diagnosed MDD (Sullivan et al., 2000). In addition, reports from family studies indicated a twofold to threefold increase in lifetime risk of MDD development among first-degree relatives (Lohoff, 2010).

Due to the inconsistencies in the results obtained from candidate gene association studies or genome-wide association studies in MDD, just like experiences with other genetically complex psychiatric disorders, no universally accepted susceptibility gene has been identified. On the other hand, many candidate genes gave hopeful preliminary results, which deserve further researching. In these candidate genes, serotonin 2A receptor (*HTR2A*) gene is said to be correlated to susceptibility in MDD (Zhang et al., 1997; Lacerda-Pinheiro et al., 2014; Zhao et al., 2014). Many subtypes of 5-HT2 receptors are found in various regions of the central nervous system, in the parts of the brain involved in emotions in particular, such as the hippocampus and the amygdala (Bombardi, 2012; Tanaka et al., 2012). As demonstrated by animal study, stress interferes with the serotonergic facilitation of GABA release mediated by 5-HT2A in the basolateral amygdala (Jiang et al., 2009). With chronic corticosterone treatment in the animal depression model, 5-HT2A receptor binding increased in the parietal cortex (Fernandes et al., 1997). One of the possible actions of antidepressant medication therapeutic mechanisms is that antidepressant medication reduces the density of 5-HT2A receptors (Carr and Lucki, 2011). 5-HT2A receptor levels are consistently reported to have increased in different brain regions of MDD patients, such as the hippocampus (Pandey et al., 2002) and the frontopolar cortex (Anisman et al., 2008) in human postmortem studies. Expression levels of *HTR2A* mRNA were increased in peripheral blood mononuclear cells of MDD, and mRNA levels of *HTR2A* itself were associated with depression severity (Amidfar et al., 2017).

The genetic effects of *HTR2A* in MDD susceptibility and antidepressant therapeutic response have been tested by many genetic studies, as 5-HT2A plays an important role in the pathogenesis of MDD as well as in the mechanism of antidepressant medication. The most frequently studied single nucleotide polymorphisms (SNPs) of the *HTR2A* gene located on 13q14.2 are A-1438G (rs6311) and T102C (rs6313) polymorphisms. Initially, Zhang et al. proposed that MDD is associated with the C allele of the rs6313 polymorphism in 1997 (Zhang et al., 1997). However, other research teams found contradictive or negative findings, despite the consistencies in replication of these results shown by some of the studies (Anguelova et al., 2003; Lin et al., 2014; Zhao et al., 2014). Likewise, there is significant association between MDD and the *HTR2A* rs6311 polymorphism (Choi et al., 2004). This is inconsistent with the results of the following studies. In 2014, a meta-analysis of 21 studies (3,299 patients and 4,092 controls) was carried out by Zhao et al. (Zhao et al., 2014). They showed that the A allele of the *HTR2A* A-1438G polymorphism might be a risk allele for MDD. On the other hand, no significant associations were found for the T102C polymorphism in MDD susceptibility. In addition, another four meta-analysis studies indicated no significant association between MDD susceptibility and the *HTR2A* polymorphisms (Anguelova et al., 2003; Lin et al., 2014; Wray et al., 2018; Howard et al., 2019).

Although the current antidepressant medications which mainly target monoamine pathways are effective, at least 30% of the MDD patients fail to achieve remission even with multiple treatment trials (Hirschfeld et al., 2002). Around 42% of the individual differences in antidepressant response were found to be explained by common genetic variants, as indicated by a sample of 2,799 antidepressant-treated MDD and genome-wide genotype data (Tansey et al., 2013). There have been inconsistent findings regarding the effects of the *HTR2A* gene on antidepressant therapeutic response, specifically, rs6311 (C > T), rs6313 (T > C), and rs7997012 (G > A, intronic SNP, residing in the second intron of the *HTR2A* gene) (Niitsu et al., 2013; Lin et al., 2014). The most frequently studied SNP out of these three *HTR2A* polymorphisms is rs7997012 for response to antidepressant medication (Niitsu et al., 2013). Within the 1,953 MDD patients in the Sequenced Treatment Alternatives for Depression study (STAR*D), rs7997012 A allele homozygotes showed better response to citalopram antidepressant medication, in comparison with homozygotes for the other allele (McMahon et al., 2006). Lin et al*.* proposed an association between therapeutic response of MDD patients to antidepressant medication and the rs7997012 G > A polymorphism under the dominant model in a meta-analysis study (Lin et al., 2014). In addition, there is a significant association with the rs6313 minor allele (C) to higher antidepressant response rate in MDD patients, but not with the rs6311 polymorphism (Lin et al., 2014).

Data interpretation of the mixed results of *HTR2A* genetic studies in MDD susceptibility and therapeutic response should be done with particular care, with regard to the complex structure of the gene, and the fact that most studies explored effects of one or several SNPs. It is possible that MDD susceptibility or antidepressant therapeutic responses may be affected by other variants in the *HTR2A* gene. Genes which may increase the vulnerability to complex diseases and therapeutic responses to medication may be detected using gene-based association tests. These tests can identify genes with genome-wide significance, but no single SNP effect is able to give genome-wide significance by univariate tests. Thus, the hypothesis that *HTR2A* may influence MDD susceptibility and antidepressant therapeutic response is verified with gene-based analysis of genome-wide data. The present study is a secondary analysis of previously published international genome-wide association study (GWAS) data (Biernacka et al., 2016). Only Taiwanese samples [MDD patients and healthy controls from Taiwan Biobank (TWB)] from two centers were used to investigate the genetic effect of *HTR2A* on antidepressant therapeutic response. A meta- and mega-analysis strategy was conducted to reduce possible false-positive results.

## Materials and Methods

### Study Subjects

A total of 455 patients were recruited in the study, who were diagnosed with MDD in two central hospitals in Taiwan, with 187 from TVGM (Taipei Veterans General Hospital) and 268 from NHRI (The National Health Research Institute) (Hong et al., 2006; Lin et al., 2010; Kao et al., 2018). Inclusion criteria were 1) diagnosis of MDD according to DSM-IV guidelines, 2) minimum baseline score of 14 on the 21-item Hamilton Rating Scale for Depression (HRSD), and 3) the presence of depressive symptoms for at least 2 weeks before entry into the study, without antidepressant treatment (patients were fresh cases or had quit antidepressants for more than 2 weeks). Exclusion criteria were additional diagnoses on Axis 1 (including schizophrenia, substance abuse, generalized anxiety disorders, panic disorders, or obsessive compulsive disorders) of the DSM-IV, personality disorders, pregnancy, attempted suicide, and major medical and/or neurological disorders. Subjects were treated with various selective serotonin reuptake inhibitors (SSRIs), including paroxetine (*n* = 162), citalopram (*n* = 20), escitalopram (*n* = 162), and fluoxetine (*n* = 77). Only three subjects were treated with sertraline, and four subjects were treated with non-SSRI (venlafaxine) and hence were excluded from the analyses to eliminate the difference from the effects of sertraline or venlafaxine. Depression severity was measured by asking all participants to answer the 21-item questionnaires of the HRSD. Patients were repetitively assessed at baseline and weeks 2, 4, and 8.

Random selection of healthy controls from a pool of 3,380 healthy individuals recruited from community subjects of the TWB with Taiwanese ancestry was made. These selected individuals were used for a case–control association study. The recruitment details were documented elsewhere (Pan et al., 2011). In short, stratified, three-stage clustering sampling design was used to recruit controls from 329 non-Aboriginal township or city districts in Taiwan. Of the 3,380 controls, 2,998 controls (nearly equal proportions of men and women) with a Taiwanese Han ancestry, who were found to have no definite diagnosis of any major medical or mental illnesses, underwent genotyping at the genome-wide level and were treated as controls in this sample. Using these MDD and control subjects, our previous report has investigated the role of ten neurotrophic-related genes in MDD susceptibility and in antidepressant efficacy (Kao et al., 2018).

Experiments were conducted in accordance with the Declaration of Helsinki and approved by the Institutional Review Board of Taipei Veterans General Hospital (VGHIRB No.: 2014-06-001B). Written informed consent forms were obtained from all participants ensuring adequate understanding of the study.

### Measurement

Four repeated phenotypes were used to measure the different treatment responses, including stem-depressed, remitted, and response. 1) Initially, the sum score of the 21-item HRSD at week 8 was calculated and recorded as 1 if the sum score is greater than 7 and recorded as 2 (i.e., remitted) otherwise. 2) We then calculated the HRSD percentage change (i.e., %ΔHRSD) at week 4 and recorded as non-response if the percentage change is less than 50% reduction in symptoms and recorded as response if otherwise. 3) We also used the percentage change of the HRSD (continuous variable) as a treatment response index. 4) For stem-depressed phenotype analysis, “depressed mood” was measured using the first item of the HRSD at week 4 and recorded as 1 if the score exceeds or is equal to 3 (i.e., severe or moderately severe) and recorded as 2 otherwise. Refer to Lin et al. for detailed definitions of the treatment response phenotypes (Lin et al., 2010).

### Multiple Imputations of Incomplete Phenotypes

Multiple imputation methods were used to solve the problem of missing phenotypes (Sterne et al., 2009). Multiple imputation is a simulation-based statistical technique, which consists of three steps: imputation, estimation, and pooling. In the first (imputation) step, missing values were estimated by fitting linear mixed regression models, based on the complete data from other variables (i.e., other phenotypic traits). The model treated all variables as fixed and all error terms as random so that every missing value was predicted by adding a random error to avoid participants with equivalent predictor values receiving the same imputed value. This guaranteed every missing value was replaced by a random sample of plausible values imputation in order to maintain the natural variability in the data. Thus, a complete dataset was created. We repeated this step 30 times (generally five times have been suggested to be sufficient on theoretical grounds) to create multiple imputed datasets (i.e., completed datasets). In the second (estimation) step, we performed standard statistical analysis (here, we used a linear mixed regression model) separately for each imputed dataset generated during the imputation step. Hereby, 30 analysis results were constructed. In the third (pooling) step, one set of results was produced by aggregating the results from each of the completed data analyses. This approach executes the above three steps for four cycles to account for uncertainty during missing data in imputation and delivered valid conclusions for downstream analysis, giving more accurate and reliable data in comparison to a single imputation. This approach maintains the overall variability in the population while preserving relationships with other variables, which guarantees important characteristics (e.g., means, variances, and regression parameters) of the data as a whole.

### Genotyping Data and Imputation

All participants were genotyped using Illumina HumanOmniExpressExome BeadChips in the International selective serotonin reuptake inhibitors (SSRI) Pharmacogenomics Consortium. A total of 455 subjects were genotyped with 951,123 SNPs. Quality control procedures were done firstly with each individual, including sample quality, kinship, and population stratification. Initially, plate-wise genotyping biases were checked. Samples with a plate pass rate greater than 97% were retained in the analysis. A total of 18 samples (11 from NHRI and 7 from TVGM) were removed during this step. Second, we checked inbreeding coefficient and identity by state so that samples with strong kinship were eliminated. A total of 9 individuals (4 from NRHI and 5 from TVGM) with measures far away from clustering were removed from the samples (Supplementary Figure S1). Third, in order to eliminate samples with outliers, we used multidimensional scaling analysis on the genome-wide identity by state pairwise distance. Results indicated that none was away from the clustering on the scatter plot (Supplementary Figure S2). Last, seven patients treated with sertraline (SSRI) or venlafaxine (SNRI) were excluded from the analysis. As a result, 421 (mean age of 43.7 years and 71.3% of women) MDD patients were retained (for details, refer to Supplementary Table S1). Quality control procedures for markers including Hardy–Weinberg tests (*p*-value > 0.0001), genotype missing rate (≤5%), minor allele frequency (MAF ≥ 0.05), and quality in clustering (good calling) were performed to the GWAS SNP data. As a result, a total of 647,030 SNPs in the samples were retained for imputation. The genotyping call rate was 99.9% for all subjects. The genotyped markers from SNP arrays were used to impute genotypes which were not observed using IMPUTE2 v3 (Howie et al., 2009), with haplotype reference panels released in March/April 2012 from the 1,000 Genomes Project on the basis of HapMap build 37 (https://mathgen.stats.ox.ac.uk/impute/data_download_1000G_phase1_integrated_SHAPEIT2.html). Only high genotype information content SNPs that were imputed (i.e., IMPUTE info score > 0.5) were included in association analyses. In total, 30,040,257 SNPs were imputed with high confidence for each individual in the samples. A total of 4,241,701 SNPs were retained for analyses following the same quality control procedures for markers. All of the 4,241,701 SNPs were mapped into 22,609 protein-coding genes using 50 kb upstream and downstream of the gene boundaries. Selection of the *HTR2A* gene variants was made, and these were mapped from the imputed genome-wide association SNP data. As a result, a total of 288 SNPs were mapped in the *HTR2A* gene.

### Difference Test

A difference test of demographic and clinical characteristics between two independent samples (i.e*.*, NHRI and TVGM) was conducted using Student’s *t*-test (for continuous data) and the chi-square test (for categorical data). The difference test of treatment response phenotypes between treatment responders and nonresponders were conducted using Student’s *t*-test (for continuous data) and the chi-square test (for categorical data).

### Genetic Association Analyses

Gene-based association and single-marker association tests were performed for treatment response phenotypes. Under the assumption of the additive genetic effect, we used linear and logistic regression models and made adjustments for age and gender to correct for distribution differences in gender and age across treatment response phenotypes in MDD patients.

Gene-based association analyses were conducted to obtain gene-level empirical significance estimation. We collected information from a set of SNPs (association *p*-value < 0.1 by default) within a gene. Only SNPs having *r* ^2^ < 0.5 with each other were retained for each gene in order to account for linkage disequilibrium (LD) among markers. Gene-based association tests were performed by fitting both the logistic regression and linear models. We summed the association signal from all SNPs that passed the above criteria within a gene and corrected the sum for LD to generate a test statistic. An empirical gene-based *p*-value was calculated with 50,000 permutation tests within each gene.

Association results across two independent samples (i.e*.*, NHRI and TVGM) were combined and summarized using the inverse gamma model with a shape parameter (α) of 1 (that is the Fisher exact method) (Zaykin et al., 2007). We followed a LD adjusted Bonferroni corrected *p*-value threshold to account for the interdependence among SNPs to balance between false-negative and false-positive findings (Duggal et al., 2008). We reported SNPs, with *p*-values less than 5 × 10^–4^ (or 5 × 10^–3^), and genes, with *p*-values less than 1 × 10^–2^ (or 5 × 10^–2^), in single-marker and gene-based association analysis, either in mega-analysis (i.e., combined two sets of independent samples, NHRI and TVGM) or meta-analysis (i.e., combined results from NHRI and TVGM), and considered to be significant (or suggestive).

All analyses were conducted with R version 3.0.2 and PLINK version 1.90b3.37 64-bit.

## Results

A summary of general demography for 428 MDD patients treated with SSRI antidepressants was shown in Table 1. In our data, our remission rate at time points of weeks 2, 4, and 8 were 6.07%, 13.32%, and 32.71%, respectively, with an overall remission rate of 36.21%. Among 428 subjects, 394 (92.06%) cases were observed at all four time points; 30 cases (7.02%) were missing at just one time point of follow-up; and the remaining four cases (0.92%) were missing at two or more time point of follow-up. Supplementary Table S2 exhibits detailed patterns of missing data in phenotypes across different time points, indicating the issue of incomplete data. Here, available data observed were used to conduct the multiple imputation method to impute these missing data so that our data were complete. Furthermore, we excluded seven subjects (four treated with sertraline and three with venlafaxine) from the analysis. As a result, 421 MDD patients were retained for the following analyses. Table 2 represents summary statistics for treatment response phenotypes. We observed that more than half (>61%) of the patients were treatment nonresponders. In comparison to 48 (11.4%) treatment nonresponders who had moderately severe or severely depressed mood, around 373 (88.6%) treatment responders were found to have moderate or less depressed mood. On the other hand, an increased response rate of taking SSRI medication from 38.72% (163 out of 421) at week 4 to 60.08% at week 8 was observed. No gender difference was observed among all treatment response phenotypes.

**TABLE 1:** Summary of general demography (*N* = 428).

	Mean (s.d.) or *N* (%)		
	Combined (*N* = 428)	NHRI (*N* = 253)	TVGM (*N* = 175)
Gender (male)	123 (28.74)	44 (17.37)	79 (45.14)
Age at time of consent (years)	43.63 (14.63)	41.00 (13.74)	47.43 (15.08)
Marriage status (married)	244 (57.01)	124 (49.01)	120 (68.57)
Number of depressive episodes	4.86 (17.99)	7.36 (23.08)	1.26 (0.60)
Suicide attempt (yes)	13 (3.04)	0 (0)	13 (7.43)
Remission rate			
Week 2	26 (6.07)	—	—
Week 4	57 (13.32)	—	—
Week 8	140 (32.71)	—	—
Antidepressant drug taken			
SSRI treatments			
Escitalopram	162 (37.85)	91 (35.97)	71 (40.57)
Paroxetine	162 (37.85)	162 (64.03)	0 (0)
Fluoxetine	77 (17.99)	0 (0)	77 (44.00)
Citalopram	20 (4.67)	0 (0)	20 (11.43)
Sertraline	3 (0.70)	0 (0)	3 (1.71)
Non-SSRI treatments			
Venlafaxine	4 (0.94)	0 (0)	4 (2.29)

s.d., standard deviation; NHRI, National Health Research Institute; TVGM, Taipei Veterans General Hospital; SSRIs, selective serotonin reuptake inhibitors. Analyses were based on 428 MDD subjects. Difference tests were conducted using Student’s *t*-test (for continuous data) or chi-square test (for categorical data). Summary statistics for age at time of consent, marriage status, and the number of depressive episodes are mean (s.d.); summary statistics for gender, suicide attempt, and antidepressant drug taken are *N* (%).

**TABLE 2:** Summary statistics for treatment response phenotypes.

Treatment response phenotype	Mean (s.d.)/*N* (%)		Gender difference *p*-value	Treatment responder			Treatment nonresponder		
	Male (*N* = 121)	Female (*N* = 300)		*N* (%)	Mean	s.d.	*N* (%)	Mean	s.d.
Remitted, score	10.36 (6.02)	11.01 (6.24)	0.32	139 (33.02)	4.46	1.93	282 (66.98)	13.96	5.03
Response, %ΔHRSD	−0.46 (0.20)	−0.42 (0.24)	0.08	163 (38.72)	−0.65	0.11	258 (61.28)	−0.29	0.17
Stem-depressed, item-wise			0.07	373 (88.60)			48 (11.40)		
Not depressed	12 (2.85)	18 (4.27)	—	30 (7.13)	—	—	0 (0.00)	—	—
Mild	54 (12.83)	115 (27.32)	—	169 (40.14)	—	—	0 (0.00)	—	—
Moderate	47 (11.16)	127 (30.16)	—	174 (41.33)	—	—	0 (0.00)	—	—
Moderately severe	7 (1.66)	38 (9.03)	—	0 (0.00)	—	—	45 (10.69)	—	—
Severe	1 (0.24)	2 (0.48)	—	0 (0.00)	—	—	3 (0.71)	—	—

s.d., standard deviation; HRSD, Hamilton Rating Scale for Depression. Analyses were based on 421 MDD subjects. Difference tests were conducted using Student’s *t*-test (for continuous data) or chi-square test (for categorical data). Summary statistics for remitted (score) and response (%ΔHRSD) are mean (s.d.); summary statistics for stem-depressed (item-wise) are *N* (%).

**FIGURE 1:**
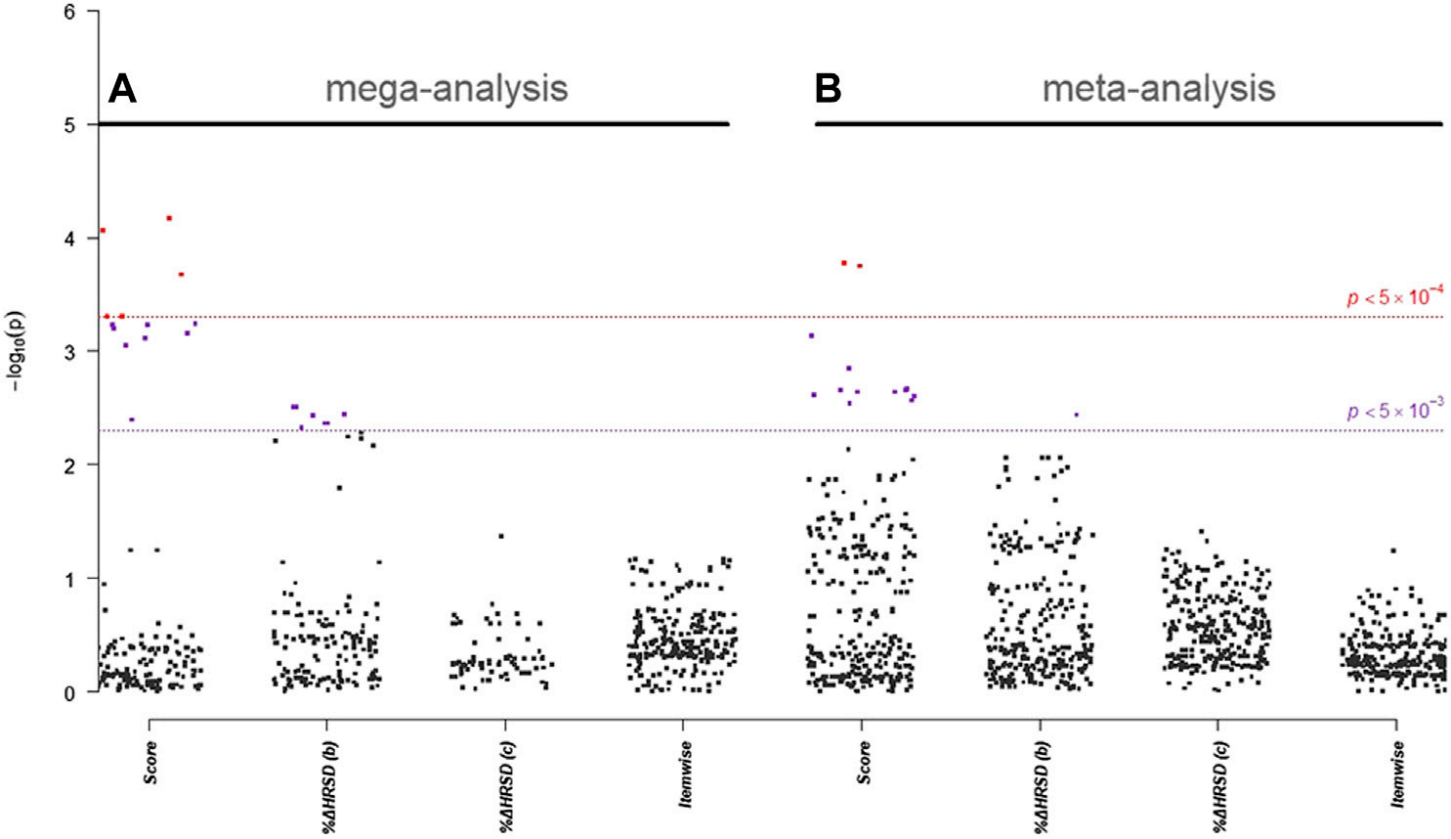
Gene plot of **(A)** mega- and **(B)** meta-analysis of *HTR2A* gene for four treatment response indices. Score represents “*remitted*”; %ΔHRSD (b) and (c) represent *response* using binary and continuous variable coding, respectively; and item-wise represents for *stem-depressed*.

In single-marker association analyses of 288 *HTR2A* SNPs, 22 SNPs with odds ratios (ORs) ranged from 0.51 to 2.21 in NHRI samples and ranged from 0.73 to 2.16 in TVGM samples (0.59–2.04 in combined samples) were reported to have suggestive signals, which reached *p*-value < 5.0 × 10^–3^ either in mega-analysis or in meta-analysis (Table 3). Among them, 13 out of 14 SNPs (mapped to *HTR2A*) were significantly (*p*-value < 5.0 × 10^–3^) associated with remission (score) in both meta-analysis and mega-analysis; one out of eight SNPs (mapped to *HTR2A*) was significantly associated with response (%ΔHRSD, binary) in both meta-analysis and mega-analysis; and none were significantly associated with stem-depressed (item-wise) in both meta-analysis and mega-analysis. For gene plots of four treatment response indices for mega- and meta-analysis, refer to Figure 1. We noted that among these 22 SNPs, only rs977003 is a chip SNP and the remaining are imputed SNPs. Results of gene-based association analyses are listed in Table 4. The *HTR2A* gene showed suggestive association (*p*-value < 5.0 × 10^–2^) in mega-analysis and/or meta-analysis in remitted (score) and in response (%ΔHRSD).

**TABLE 3:** *HTR2A* susceptibility loci for major depressive disorder that related to response to antidepressant drug treatment in Han Chinese.

SNP	CHR: Position	Intron	NHRI			TVGM			Mega-analysis			Meta-analysis *p*-value
			MAF	OR/$\hat{\beta }$	*p*-value	MAF	OR/$\hat{\beta }$	*p*-value	MAF	OR/$\hat{\beta }$	*p*-value	
Remitted (score, binary)												
rs7333412	13:47403360	Downstream	0.1779	1.91	6.1 × 10^–3^	0.1518	2.00	5.2 × 10^–2^	0.1659	1.88	**9.0 × 10** ^**–4**^	**2.9 × 10** ^**–3**^
rs7324017	13:47406845	3′UTR	0.1779	1.91	6.1 × 10^–3^	0.1488	2.05	4.4 × 10^–2^	0.1647	1.91	**7.1 × 10** ^**–4**^	**2.5 × 10** ^**–3**^
rs3803189	13:47408570	3′UTR	0.1779	1.91	6.1 × 10^–3^	0.1467	2.10	3.9 × 10^–2^	0.1639	1.93	**5.9 × 10** ^**–4**^	**2.2 × 10** ^**–3**^
rs3125	13:47408851	3′UTR	0.1779	1.91	6.1 × 10^–3^	0.1467	2.10	3.9 × 10^–2^	0.1639	1.93	**5.9 × 10** ^**–4**^	**2.2 × 10** ^**–3**^
rs7322347	13:47410103	Intron 3	0.2321	2.19	5.3 × 10^–4^	0.2024	2.10	2.6 × 10^–2^	0.2190	2.04	**6.9 × 10** ^**–4**^	**1.7 × 10** ^**–4**^
chr13:47410325:D	13:47410325	Intron 3	0.1844	1.91	6.7 × 10^–3^	0.1509	2.16	3.5 × 10^–2^	0.1695	1.95	**5.8 × 10** ^**–4**^	**2.2 × 10** ^**–3**^
rs1923882	13:47411661	Intron 3	0.1786	1.90	6.8 × 10^–3^	0.1488	2.05	4.4 × 10^–2^	0.1651	1.90	**7.7 × 10** ^**–4**^	**2.7 × 10** ^**–3**^
rs55948462	13:47412075	Intron 3	0.1786	1.90	6.8 × 10^–3^	0.1467	2.10	3.9 × 10^–2^	0.1643	1.92	**6.4 × 10** ^**–4**^	**2.4 × 10** ^**–3**^
rs56005991	13:47412741	Intron 3	0.1873	2.18	1.1 × 10^–3^	0.1647	1.89	6.3 × 10^–2^	0.1775	2.01	**2.1 × 10** ^**–4**^	**7.4 × 10** ^**–4**^
rs977003	13:47415001	Intron 3	0.2312	2.21	4.5 × 10^–4^	0.2054	2.01	3.3 × 10^–2^	0.2209	2.00	**8.8 × 10** ^**–5**^	**1.8 × 10** ^**–4**^
rs61948314	13:47415383	Intron 3	0.1714	1.95	5.9 × 10^–3^	0.1402	2.10	4.2 × 10^–2^	0.1575	1.98	**4.9 × 10** ^**–4**^	**2.3 × 10** ^**–3**^
rs75907607	13:47416042	Intron 3	0.1714	1.95	5.9 × 10^–3^	0.1402	2.10	4.2 × 10^–2^	0.1575	1.98	**4.9 × 10** ^**–4**^	**2.3 × 10** ^**–3**^
rs17068986	13:47416386	Intron 3	0.4822	0.51	5.2 × 10^–4^	0.4940	0.73	2.8 × 10^–1^	0.4883	0.59	**6.0 × 10** ^**–4**^	**1.2 × 10** ^**–3**^
rs76703096	13:47416712	Intron 3	0.1390	1.84	2.5 × 10^–2^	0.1180	1.97	8.5 × 10^–2^	0.1296	1.86	**4.1 × 10** ^**–3**^	1.5 × 10^–2^
Response (%ΔHRSD, binary)												
rs7333412	13:47403360	Downstream	0.1779	1.72	2.0 × 10^–2^	0.1518	1.86	5.6 × 10^–2^	0.1659	1.72	**3.7 × 10** ^**–3**^	8.7 × 10^–3^
rs3803189	13:47408570	3′UTR	0.1779	1.72	2.0 × 10^–2^	0.1467	1.80	7.1 × 10^–2^	0.1639	1.71	**4.4 × 10** ^**–3**^	1.1 × 10^–2^
rs3125	13:47408851	3′UTR	0.1779	1.72	2.0 × 10^–2^	0.1467	1.80	7.1 × 10^–2^	0.1639	1.71	**4.4 × 10** ^**–3**^	1.1 × 10^–2^
chr13:47410325:D	13:47410325	Intron 3	0.1844	1.69	2.6 × 10^–2^	0.1509	1.97	4.3 × 10^–2^	0.1695	1.74	**3.6 × 10** ^**–3**^	8.8 × 10^–3^
rs55948462	13:47412075	Intron 3	0.1786	1.71	2.2 × 10^–2^	0.1467	1.80	7.1 × 10^–2^	0.1643	1.70	**4.7 × 10** ^**–3**^	1.2 × 10^–2^
rs61948314	13:47415383	Intron 3	0.1714	1.75	1.9 × 10^–2^	0.1402	1.88	5.9 × 10^–2^	0.1575	1.77	**3.1 × 10** ^**–3**^	8.9 × 10^–3^
rs75907607	13:47416042	Intron 3	0.1714	1.75	1.9 × 10^–2^	0.1402	1.88	5.9 × 10^–2^	0.1575	1.77	**3.1 × 10** ^**–3**^	8.9 × 10^–3^
rs17068986	13:47416386	Intron 3	0.4822	0.54	1.4 × 10^–3^	0.4940	0.78	3.0 × 10^–1^	0.4883	0.62	**1.1 × 10** ^**–3**^	**3.3 × 10** ^**–3**^

SNP, single nucleotide polymorphism; CHR, chromosome; MAF, minor allele frequency; OR, odds ratio; NHRI, National Health Research Institute; TVGM, Taipei Veterans General Hospital; HRSD, Hamilton Rating Scale for Depression. Association analyses were based on 421 MDD subjects, using linear (or logistic) regression with an additive model (1-degree of freedom) after adjusting for gender and age. Meta-analysis p-value was calculated using the inverse gamma model with a shape parameter (α) of 1 (that is the Fisher exact method). Only SNPs that reached p-value of less than 5 × 10^–4^, 5 × 10^–3^, or 1 × 10^–2^ either in mega-analysis or in meta-analysis were reported as strong, suggestive, or weak significant markers. SNPs highlighted in bold are chip markers. Significance for bold entries represents significant (or suggestive) loci either in mega‐analysis or in meta‐analysis.

**TABLE 4:** *HTR2A* susceptibility genes for major depressive disorder that related to response to antidepressant drug treatment in Han Chinese.

Gene	Pathway related to antidepressant effect	NHRI		TVGM		Mega-analysis		Meta-analysis *p*-value
		Prop	*p*-value	Prop	*p*-value	Prop	*p*-value	
Remitted (score, binary)								
*HTR2A*	Serotonin system	31/275	9.2 × 10^–3^	22/275	3.7 × 10^–1^	20/275	**6.6 × 10** ^**–4**^	**2.0 × 10** ^**–2**^
Response (%ΔHRSD, binary)								
*HTR2A*	Serotonin system	17/275	1.5 × 10^–1^	51/275	1.9 × 10^–1^	21/275	**4.9 × 10** ^**–3**^	1.3 × 10^–1^

Prop, proportion of significant SNPs; NHRI, National Health Research Institute; TVGM, Taipei Veterans General Hospital; HRSD, Hamilton Rating Scale for Depression. Gene-based association analyses were based on 421 major depressive disorder patients. Only genes that reached *p*-value < 0.01 (or 0.05) either in mega-analysis or in meta-analysis were reported as significant (or suggestive) loci, based on 50,000 permutations. Models were adjusted for gender and age. Proportion of significant SNPs was defined as the number of significant SNPs (*p*-value < 0.05) divided by total number of SNPs in that gene. We conducted linear (if continuous) and logistic (if dichotomous) regressions analyses for treatment response phenotypes. Significance for bold entries represents significant (or suggestive) loci either in mega-analysis or in meta-analysis.

We also conducted a case–control association study using samples from NHRI and TVGH (421 MDD patients) and TWB (2,998 healthy controls). All reported genes and SNPs reported did not reach a significant association level. (Refer to Supplementary Table S3 for detailed information on single-marker association and Supplementary Table S4 for gene-based association details.) Note that we only reported available SNPs extracted from two assays of GWAS genotypes in both case samples (NHRI and TVGM) and healthy control samples (HCCGB).

## Discussion

In this study, our remission rate at weeks 2, 4, and 8 were 6.07%, 13.32%, and 32.71%, respectively, with an overall remission rate of 36.21%. Our results were similar to the remission rates (38.9%) of the STAR*D study (Trivedi et al., 2006). Furthermore, around 39% of the patients were found to show response after 4 weeks of taking SSRI medication. These results suggested similar remission and response rates, which echoed the results of a review article by Kennedy (2013). On the other hand, an increased response rate from 38.72% at week 4 to 60.08% at 8 weeks was observed, showing a higher rate of response than the 52.4% at week 12 by Rush et al.’s STAR*D study (Rush et al., 2011).

In our single-marker association analyses, 14 SNPs (where eight SNPs were reported in both remission and response) were reported to have suggestive signals either in mega-analysis or in meta-analysis. Using samples from NHRI, TVGM, as well as a combination of both, 13 SNPs (where rs17068986 SNP was reported in both remission and response) out of the 14 *HTR2A* SNPs were found to be suggestive markers for treatment response phenotypes (13 for remission and one for response). Only rs977003 is a chip SNP, and the remaining 13 SNPs are imputed SNPs. In the positron emission tomography (PET) study, rs977003 appears to be significantly associated with serotonin transporter binding potential (Laje et al., 2010). This polymorphism, as found by recent study, is able to moderate the association between reduced default mode network connectivity and severity of post-traumatic stress disorder (Miller et al., 2016). Therefore, modulation of antidepressant binding on the serotonin transporter or brain circuits by rs977003 may influence the therapeutic response of the SSRI medication. The remaining 13 imputed SNPs provide a novel association for further replication studies. The importance of genotype imputation in this study include 1) increasing the density of genotype calls for fine-mapping to identify genetic loci that contribute susceptibility for treatment response phenotypes, 2) boosting statistical power, and 3) harmonization of different GWAS genotyping platforms for conducting meta- and mega-analysis.

For other *HTR2A* SNPs related to MDD therapeutic response, MDD patients with the rs7333412 GG genotype have been reported to be less prone to antidepressants than patients of A allele carriers (Qesseveur et al., 2016). PET measurements indicated that this polymorphism is also able to affect serotonin transporter binding potential (Laje et al., 2010). In a similar way to rs977003, rs7322347 regulates the association between decreased default mode network connectivity and severity of post-traumatic stress disorder (Miller et al., 2016). An association between suicide attempt in schizophrenia patients and rs17068986 has been reported (Bani-Fatemi et al., 2016). We used HaploReg to predict a possible functional role of these 13 *HTR2A* SNPs. We found that seven SNPs (rs7333412, rs7324017, rs7322347, rs1923882, rs56005991, rs977003, and rs17068986) exhibited direct eQTL effects. Of note, functional annotation by HaploReg indicated that transcriptional regulation activity exists at the seven SNP loci for the *HTR2A* gene in peripheral blood monocytes, dendritic cells, prefrontal cortex, blood, and/or breast tumors. (Refer to Supplementary Table S5 for detailed information on eQTL prediction for the seven SNPs in the *HTR2A* gene.) With *Htr2a* knockout mutants in animal studies, chronic administration of antidepressant failed to produce antidepressant-like activity (Quesseveur et al., 2013; Qesseveur et al., 2016). Therefore, these *HTR2A* polymorphisms may affect *HTR2A* expression and result in different responses to antidepressant treatments. Further experimental investigations are required to elucidate the functional impacts of these polymorphisms in antidepressant mechanisms.

Although previous studies have proposed that rs6313, rs7997012, and rs6311 may be related to antidepressant therapeutic response, replication studies indicated inconsistencies in such results (Niitsu et al., 2013; Yan et al., 2015; Nuntamool et al., 2017; Alladi et al., 2018). These three SNPs found in our analysis of 288 *HTR2A* SNPs were not associated with antidepressant therapeutic phenotypes in our GWAS (for details, refer to Supplementary Table S6).

As the *HTR2A* gene plays the key role in serotonergic function, it is said to be correlated with MDD susceptibility. In 1997, the number of rs6313 C alleles was first reported to be significantly higher in MDD patients than in healthy control subjects (Zhang et al., 1997). The results of the following studies, however, have been inconsistent, and other *HTR2A* polymorphisms have also been investigated in MDD susceptibility (Anguelova et al., 2003; Lin et al., 2014; Zhao et al., 2014). The second aim of this study was to investigate the *HTR2A* genetic effect on MDD susceptibility. We conducted the case–control association study using 421 MDD patients and 2,998 healthy controls with 288 *HTR2A* SNPs. All the *HTR2A* gene SNPs reported did not reach a significant level in terms of quantity in the analysis, indicating that the *HTR2A* gene may be specifically associated with SSRI treatment response but not MDD susceptibility. As in support with the previous meta-analysis of *HTR2A* genetic association studies in MDD (Anguelova et al., 2003; Lin et al., 2014), our study does not indicate *HTR2A* polymorphisms as major role players in the contribution to MDD susceptibility.

This study applied gene-set association through analyses of pharmacogenetics association (i.e., treatment response–based association in a case-only study) and disease association (i.e., diagnosis-based association in a case–control study) to identify if *HTR2A* polymorphisms had an effect on SSRI antidepressant treatment response. The strengths of this study include 1) large cohort with treatment outcomes and 2) large cohort with medication and genotyping. However, this study has some limitations that should be addressed. First, case controls were not matched. In our study, we corrected relatedness and population structure and adjusted for sex and age in the analyses to balance out possible confounders. Second, the two patient cohorts were significantly different in demographics and treatment history. Third, since the data were analyzed with various SSRIs, they did not allow analyses to focus on specific drugs. Fourth, this study is only focused on the *HTR2A* gene. Further studies of other of serotonin-related genes are required.

## Conclusion

In summary, our gene-based association analysis found 14 (out of 288) *HTR2A* SNPs that are associated with SSRI therapeutic phenotypes in both meta-analysis and mega-analysis from two MDD groups, but no association with MDD susceptibility was found. As expected for a single gene, the clinical impact of *HTR2A* on treatment outcome is modest. The power to detect loci with the observed effect sizes in our mega-analysis and meta-analysis is modest, ranging from 0.30–0.53 and 0.39–0.42, respectively, given a disease prevalence of 1–5%. Further replication studies in a larger sample are needed to confirm our findings and find other antidepressant therapeutic related genetic variants.

## Data Availability Statement

The datasets generated for this study can be found in EVA, https://www.ebi.ac.uk/ena/data/view/PRJEB40684

## Author Contributions

C-FK designed analytical frameworks of data and performed data management and quality control and statistical analyses, wrote most parts of the paper, and revised the manuscript. Study conception and design: EL, AY, YY, and S-JT; acquisition of data: P-HK, Y-LL, and C-FK; analysis and interpretation of data: C-FK, P-HK, and S-JT; draft and revise manuscript: C-FK, P-HK, and S-JT. All authors read and approved the final manuscript.

## Funding

Genotyping of MDD subjects was funded by the RIKEN Center for Integrative Medical Science, Yokohama, Japan. This work was supported by grant MOST 109-2634-F-075-001 from Taiwan Ministry of Science and Technology, and grant V108D44-001-MY3-1 from the Taipei Veterans General Hospital. This work was financially supported (in part) by the Advanced Plant Biotechnology Center from The Featured Areas Research Center Program within the framework of the Higher Education Sprout Project by the Ministry of Education (MOE) in Taiwan.

## Conflict of Interest

The authors declare that the research was conducted in the absence of any commercial or financial relationships that could be construed as a potential conflict of interest.
