# Supplementary material for: Gene-Based Association Analysis Suggests Association of HTR2A With Antidepressant Treatment Response in Depressed Patients
Source: Front Pharmacol. 2020 Dec 3;11:559601. doi: 10.3389/fphar.2020.559601 (PMC7845659; doi:10.3389/fphar.2020.559601)

**Supporting information**

**Table S1**

Summary of data quality control of subjects.

|  | Original samples | Plate QC check Call rate <97% | Kinship | Population stratification | SSRIs treatment |
| --- | --- | --- | --- | --- | --- |
| NHRI | 268 | 257 | 253 | 253 | 253 |
| TVGM | 187 | 180 | 175 | 175 | 168 |
| Total | 455 | 437 | 428 | 428 | 421 |
| Abbreviation: QC, quality control. SSRIs, selective serotonin reuptake inhibitors. | | | | | |

**Table S2**

The patterns of missing data in phenotypes (N=428).

| Time point | Missing data patterns | | | | | | | # of missing observation (%) |
| --- | --- | --- | --- | --- | --- | --- | --- | --- |
| Baseline | Observed | Observed | Observed | Observed | Observed | Observed | Observed | 0 |
| Follow-up (week 2) | Observed | **Missing** | Observed | Observed | **Missing** | **Missing** | **Missing** | 14 (3.27%) |
| Follow-up (week 4) | Observed | Observed | **Missing** | Observed | **Missing** | Observed | **Missing** | 12 (2.80%) |
| Follow-up (week 8) | Observed | Observed | Observed | **Missing** | Observed | **Missing** | **Missing** | 13 (3.04%) |
| # of cases | 394 | 10 | 10 | 10 | 1 | 2 | 1 | 428 |
| (%) | (92.06%) | (2.34%) | (2.34%) | (2.34%) | (0.23%) | (0.46%) | (0.23%) |  |

**Table S3**

Association results of significant markers in a case-control study of MDD.

| SNP | CHR: Position | Mapped gene | MAF | |  | Association | |
| --- | --- | --- | --- | --- | --- | --- | --- |
|  |  |  | Cases | Controls |  | OR | *p*-value |
| rs7324017 | 13:47406845 | *HTR2A* | 0.1659 | 0.1768 |  | 0.92 | 0.39 |
| rs61948314 | 13:47415383 |  | 0.1575 | 0.1760 |  | 0.88 | 0.19 |
| rs17068986 | 13:47416386 |  | 0.4883 | 0.4766 |  | 1.05 | 0.51 |
| rs76703096 | 13:47416712 |  | 0.1296 | 0.1448 |  | 0.88 | 0.24 |
| Abbreviation: SNP, single nucleotide polymorphism; CHR, chromosome; MAF, minor allele frequency; OR, odds ratio.  ^a^ Association analyses were based on 3419 subjects (421 MDD patients and 2998 controls), using logistic regression with an additive model (1-degree of freedom). ^b^ Only 22 SNPs that reached *p*-value<5.0×10^-3^ either in mega-analysis or in meta-analysis were conducted for case-control association; among them, only 4 SNPs provided genotypes information in both cases and controls. | | | | | | | |

**Table S4**

Results of gene-based association analyses for MDD.

| Gene | Number of SNPs within a gene |  | Number of SNPs having *p*-value<0.1 |  | Number of SNPs having *p*-value<0.1 and *r*^2^<0.5 | Empirical *p*-value |
| --- | --- | --- | --- | --- | --- | --- |
| *HTR2A* | 288 |  | 2 |  | 2 | 0.86 |
| Abbreviation: SNP, single nucleotide polymorphism.  ^a^ Association analyses were based on 3419 subjects (421 MDD patients and 2998 controls), using 100,000 permutations in gene-based association analysis. | | | | | | |

**Table S5**

Summary of eQTL prediction.

**(a) Query SNPs: rs7333412 and variants with r^2^≥0.8 and eQTL h**its

| Chr | Position | LD (r^2^) | LD (D’) | Variant | Ref | Alt | EUR freq | Enhancer histone marks | DNAse | Proteins bound | Motifs changed | GRASP QTL hits | Selected eQTL hits | GENCODE genes |
| --- | --- | --- | --- | --- | --- | --- | --- | --- | --- | --- | --- | --- | --- | --- |
| 13 | 46829225 | 1 | 1 | [**rs7333412**](https://pubs.broadinstitute.org/mammals/haploreg/detail_v4.1.php?query=&id=rs7333412) | A | G | 0.21 | LIV |  |  | HDAC2, Myc | 2 hits |  | 4.2kb 3' of HTR2A |
| 13 | 46832710 | 0.99 | 1 | [rs7324017](https://pubs.broadinstitute.org/mammals/haploreg/detail_v4.1.php?query=&id=rs7324017) | C | T | 0.21 | FAT | BRN |  | CEBPG, ZEB1 | 1 hit |  | 667bp 3' of HTR2A |
| 13 | 46837526 | 0.99 | 1 | [rs1923882](https://pubs.broadinstitute.org/mammals/haploreg/detail_v4.1.php?query=&id=rs1923882) | C | T | 0.21 |  |  |  | 4 altered motifs | 1 hit |  | HTR2A |
| 13 | 46838606 | 0.92 | 0.99 | [rs56005991](https://pubs.broadinstitute.org/mammals/haploreg/detail_v4.1.php?query=&id=rs56005991) | G | A | 0.22 |  |  |  | 6 altered motifs |  |  | HTR2A |

**(b) Query SNPs: rs7324017 and variants with r^2^**≥0.8 and eQTL hits

| Chr | Position | LD (r^2^) | LD (D’) | Variant | Ref | Alt | | EUR freq | Enhancer histone marks | DNAse | Proteins bound | Motifs changed | GRASP QTL hits | Selected eQTL hits | GENCODE genes | |
| --- | --- | --- | --- | --- | --- | --- | --- | --- | --- | --- | --- | --- | --- | --- | --- | --- |
| 13 | 46829225 | 0.99 | 1 | [rs7333412](https://pubs.broadinstitute.org/mammals/haploreg/detail_v4.1.php?query=&id=rs7333412) | A | G | | 0.21 | LIV |  |  | HDAC2, Myc | 2 hits |  | 4.2kb 3' of HTR2A | |
| 13 | 46832710 | 1 | 1 | [**rs7324017**](https://pubs.broadinstitute.org/mammals/haploreg/detail_v4.1.php?query=&id=rs7324017) | C | | T | 0.21 | FAT | BRN |  | CEBPG, ZEB1 | 1 hit |  | | 667bp 3' of HTR2A |
| 13 | 46837526 | 1 | 1 | [rs1923882](https://pubs.broadinstitute.org/mammals/haploreg/detail_v4.1.php?query=&id=rs1923882) | C | | T | 0.21 |  |  |  | 4 altered motifs | 1 hit |  | | HTR2A |
| 13 | 46838606 | 0.93 | 0.99 | [rs56005991](https://pubs.broadinstitute.org/mammals/haploreg/detail_v4.1.php?query=&id=rs56005991) | G | A | | 0.22 |  |  |  | 6 altered motifs |  |  | HTR2A | |

**(c) Query SNPs: rs7322347 and variants with r^2^**≥0.8 and eQTL hits

| Chr | Position | LD (r^2^) | LD (D’) | Variant | Ref | Alt | EUR freq | Enhancer histone marks | DNAse | Proteins bound | Motifs changed | GRASP QTL hits | Selected eQTL hits | GENCODE genes |
| --- | --- | --- | --- | --- | --- | --- | --- | --- | --- | --- | --- | --- | --- | --- |
| 13 | 46835968 | 1 | 1 | [**rs7322347**](https://pubs.broadinstitute.org/mammals/haploreg/detail_v4.1.php?query=&id=rs7322347) | T | A | 0.43 |  |  |  | CEBPB, Dbx1, Sox |  | 1 hit | HTR2A |
| 13 | 46840866 | 0.98 | 1 | [rs977003](https://pubs.broadinstitute.org/mammals/haploreg/detail_v4.1.php?query=&id=rs977003) | A | C | 0.44 |  |  | CHD2 | 5 altered motifs | 4 hits | 1 hit | HTR2A |

**(d) Query SNPs: rs1923882 and variants with r^2^**≥0.8 and eQTL hits

| Chr | Position | LD (r^2^) | LD (D’) | Variant | Ref | Alt | | EUR freq | Enhancer histone marks | DNAse | Proteins bound | Motifs changed | GRASP QTL hits | Selected eQTL hits | GENCODE genes | |
| --- | --- | --- | --- | --- | --- | --- | --- | --- | --- | --- | --- | --- | --- | --- | --- | --- |
| 13 | 46829225 | 0.99 | 1 | [rs7333412](https://pubs.broadinstitute.org/mammals/haploreg/detail_v4.1.php?query=&id=rs7333412) | A | G | | 0.21 | LIV |  |  | HDAC2, Myc | 2 hits |  | 4.2kb 3' of HTR2A | |
| 13 | 46832710 | 1 | 1 | [rs7324017](https://pubs.broadinstitute.org/mammals/haploreg/detail_v4.1.php?query=&id=rs7324017) | C | | T | 0.21 | FAT | BRN |  | CEBPG, ZEB1 | 1 hit |  | | 667bp 3' of HTR2A |
| 13 | 46837526 | 1 | 1 | [**rs1923882**](https://pubs.broadinstitute.org/mammals/haploreg/detail_v4.1.php?query=&id=rs1923882) | C | | T | 0.21 |  |  |  | 4 altered motifs | 1 hit |  | | HTR2A |
| 13 | 46838606 | 0.93 | 1 | [rs56005991](https://pubs.broadinstitute.org/mammals/haploreg/detail_v4.1.php?query=&id=rs56005991) | G | A | | 0.22 |  |  |  | 6 altered motifs |  |  | HTR2A | |

**(e) Query SNPs: rs56005991 and variants with r^2^**≥0.8 and eQTL hits

| Chr | Position | LD (r^2^) | LD (D’) | Variant | Ref | Alt | | EUR freq | Enhancer histone marks | DNAse | Proteins bound | Motifs changed | GRASP QTL hits | Selected eQTL hits | GENCODE genes | |
| --- | --- | --- | --- | --- | --- | --- | --- | --- | --- | --- | --- | --- | --- | --- | --- | --- |
| 13 | 46829225 | 0.92 | 0.99 | [rs7333412](https://pubs.broadinstitute.org/mammals/haploreg/detail_v4.1.php?query=&id=rs7333412) | A | G | | 0.21 | LIV |  |  | HDAC2, Myc | 2 hits |  | 4.2kb 3' of HTR2A | |
| 13 | 46832710 | 0.93 | 1 | [rs7324017](https://pubs.broadinstitute.org/mammals/haploreg/detail_v4.1.php?query=&id=rs7324017) | C | | T | 0.21 | FAT | BRN |  | CEBPG, ZEB1 | 1 hit |  | | 667bp 3' of HTR2A |
| 13 | 46837526 | 0.93 | 1 | [rs1923882](https://pubs.broadinstitute.org/mammals/haploreg/detail_v4.1.php?query=&id=rs1923882) | C | | T | 0.21 |  |  |  | 4 altered motifs | 1 hit |  | | HTR2A |
| 13 | 46838606 | 1 | 1 | [**rs56005991**](https://pubs.broadinstitute.org/mammals/haploreg/detail_v4.1.php?query=&id=rs56005991) | G | A | | 0.22 |  |  |  | 6 altered motifs |  |  | HTR2A | |

**(f) Query SNPs: rs977003 and variants with r^2^**≥0.8 and eQTL hits

| Chr | Position | LD (r^2^) | LD (D’) | Variant | Ref | Alt | EUR freq | Enhancer histone marks | DNAse | Proteins bound | Motifs changed | GRASP QTL hits | Selected eQTL hits | GENCODE genes |
| --- | --- | --- | --- | --- | --- | --- | --- | --- | --- | --- | --- | --- | --- | --- |
| 13 | 46835968 | 0.98 | 1 | [rs7322347](https://pubs.broadinstitute.org/mammals/haploreg/detail_v4.1.php?query=&id=rs7322347) | T | A | 0.43 |  |  |  | CEBPB, Dbx1, Sox |  | 1 hit | HTR2A |
| 13 | 46840866 | 1 | 1 | [**rs977003**](https://pubs.broadinstitute.org/mammals/haploreg/detail_v4.1.php?query=&id=rs977003) | A | C | 0.44 |  |  | CHD2 | 5 altered motifs | 4 hits | 1 hit | HTR2A |

**(g) Query SNPs: rs17068986 and variants with r^2^**≥0.8 and eQTL hits

| Chr | Position | LD (r^2^) | LD (D’) | Variant | Ref | Alt | | EUR freq | Enhancer histone marks | DNAse | Proteins bound | Motifs changed | GRASP QTL hits | Selected eQTL hits | GENCODE genes | |
| --- | --- | --- | --- | --- | --- | --- | --- | --- | --- | --- | --- | --- | --- | --- | --- | --- |
| 13 | 46842251 | 1 | 1 | [**rs17068986**](https://pubs.broadinstitute.org/mammals/haploreg/detail_v4.1.php?query=&id=rs17068986) | C | T | | 0.12 |  |  |  | 6 altered motifs |  | 1 hit | HTR2A | |
| 13 | 46845069 | 0.93 | 0.97 | [rs9567735](https://pubs.broadinstitute.org/mammals/haploreg/detail_v4.1.php?query=&id=rs9567735) | A | | G | 0.12 |  |  |  |  | 1 hit | 1 hit | | HTR2A |
| 13 | 46845442 | 0.91 | 0.96 | [rs3742278](https://pubs.broadinstitute.org/mammals/haploreg/detail_v4.1.php?query=&id=rs3742278) | A | | G | 0.12 | ESDR | ESDR, SKIN | GATA2 | 4 altered motifs |  | 1 hit | | HTR2A |
| 13 | 46846277 | 0.93 | 0.97 | [rs9562684](https://pubs.broadinstitute.org/mammals/haploreg/detail_v4.1.php?query=&id=rs9562684) | A | | C | 0.12 |  |  |  | 10 altered motifs |  | 1 hit | | HTR2A |
| 13 | 46846848 | 0.91 | 0.96 | [rs9567736](https://pubs.broadinstitute.org/mammals/haploreg/detail_v4.1.php?query=&id=rs9567736) | G | | A | 0.12 | MUS |  |  |  |  |  | | HTR2A |
| 13 | 46847088 | 0.93 | 0.97 | [rs9562685](https://pubs.broadinstitute.org/mammals/haploreg/detail_v4.1.php?query=&id=rs9562685) | G | | A | 0.12 | MUS, CRVX |  |  | CAC-binding-protein, Pax-4, SETDB1 |  | 1 hit | | HTR2A |
| 13 | 46847701 | 0.93 | 0.97 | [rs1923884](https://pubs.broadinstitute.org/mammals/haploreg/detail_v4.1.php?query=&id=rs1923884) | C | T | | 0.12 | BLD | ESC |  |  |  | 1 hit | HTR2A | |

**Table S6**

No association between antipsychotics response and rs6313, rs631 and rs7997012 of *HTR2A* polymorphisms for major depressive disorder in Han Chinese.

| SNP | | CHR: Position | Intron |  | NHRI | | |  | TVGM | | |  | Mega-analysis | | |  | Meta-  analysis *p*-value |
| --- | --- | --- | --- | --- | --- | --- | --- | --- | --- | --- | --- | --- | --- | --- | --- | --- | --- |
|  |  |  |  |  | MAF | OR/$\hat{\beta}$ | *p*-value |  | MAF | OR/$\hat{\beta}$ | *p*-value |  | MAF | OR/$\hat{\beta}$ | *p*-value |  |  |
|  | **Remitted (Score, binary)** | | | | | | | | | | | | | | | | |
| rs7997012 | | 13:47411985 | intron |  | 0.2857 | 1.12 | 5.6×10^-1^ |  | 0.2994 | 0.81 | 4.9×10^-1^ |  | 0.2911 | 1.03 | 8.8×10^-1^ |  | 6.3×10^-1^ |
| rs6313 | | 13:47469940 | intron |  | 0.3972 | 1.31 | 1.5×10^-1^ |  | 0.4137 | 0.71 | 1.9×10^-1^ |  | 0.4019 | 1.04 | 8.1×10^-1^ |  | 1.3×10^-1^ |
| rs6311 | | 13:47471478 | upstream |  | 0.3972 | 1.31 | 1.5×10^-1^ |  | 0.4137 | 0.71 | 1.9×10^-1^ |  | 0.4019 | 1.04 | 8.1×10^-1^ |  | 1.3×10^-1^ |
|  | **Response (%ΔHRSD, binary)** | | | | | | | | | | | | | | | | |
| rs7997012 | | 13:47411985 | intron |  | 0.2857 | 1.35 | 1.3×10^-1^ |  | 0.2994 | 0.89 | 6.3×10^-1^ |  | 0.2911 | 1.18 | 2.7×10^-1^ |  | 2.8×10^-1^ |
| rs6313 | | 13:47469940 | intron |  | 0.3972 | 1.27 | 2.1×10^-1^ |  | 0.4137 | 0.91 | 6.5×10^-1^ |  | 0.4019 | 1.10 | 4.9×10^-1^ |  | 4.1×10^-1^ |
| rs6311 | | 13:47471478 | upstream |  | 0.3972 | 1.27 | 2.1×10^-1^ |  | 0.4137 | 0.91 | 6.5×10^-1^ |  | 0.4019 | 1.10 | 4.9×10^-1^ |  | 4.1×10^-1^ |
|  | **Response (%ΔHRSD, continuous)** | | | | | | | | | | | | | | | | |
| rs7997012 | | 13:47411985 | intron |  | 0.2857 | -0.04 | 5.3×10^-2^ |  | 0.2994 | 0.02 | 3.5×10^-1^ |  | 0.2911 | -0.02 | 1.8×10^-1^ |  | 9.3×10^-2^ |
| rs6313 | | 13:47469940 | intron |  | 0.3972 | -0.04 | 9.7×10^-2^ |  | 0.4137 | -0.01 | 7.5×10^-1^ |  | 0.4019 | -0.03 | 1.1×10^-1^ |  | 2.6×10^-1^ |
| rs6311 | | 13:47471478 | upstream |  | 0.3972 | -0.04 | 9.7×10^-2^ |  | 0.4137 | -0.01 | 7.5×10^-1^ |  | 0.4019 | -0.03 | 1.1×10^-1^ |  | 2.6×10^-1^ |
|  | **Stem-depressed (Item-wise, binary)** | | | | | | | | | | | | | | | | |
| rs7997012 | | 13:47411985 | intron |  | 0.2857 | 1.91 | 5.1×10^-2^ |  | 0.2994 | 1.01 | 9.8×10^-1^ |  | 0.2911 | 1.55 | 8.6×10^-2^ |  | 2.0×10^-1^ |
| rs6313 | | 13:47469940 | intron |  | 0.3972 | 1.29 | 3.6×10^-1^ |  | 0.4137 | 1.21 | 6.1×10^-1^ |  | 0.4019 | 1.29 | 2.6×10^-1^ |  | 5.5×10^-1^ |
| rs6311 | | 13:47471478 | upstream |  | 0.3972 | 1.29 | 3.6×10^-1^ |  | 0.4137 | 1.21 | 6.1×10^-1^ |  | 0.4019 | 1.29 | 2.6×10^-1^ |  | 5.5×10^-1^ |
|  | Abbreviation: SNP, single nucleotide polymorphism; CHR, chromosome; MAF, minor allele frequency; OR, odds ratio; NHRI, National Health Research Institute; TVGM, Taipei Veterans General Hospital; HRSD, Hamilton Rating Scale for Depression.  Association analyses were based on 421 MDD subjects, using linear (or logistic) regression with an additive model (1-degree of freedom) after adjusting for gender and age. Meta-analysis *p*-value was calculated using the inverse Gamma model with a shape parameter (α) of 1 (that is the Fisher exact method). Only SNPs that reached *p*-value of less than 5×10^-4^, 5×10^-3^ or 1×10^-2^ either in mega-analysis or in meta-analysis were reported as strong, suggestive or weak significant markers. SNPs highlighted in bold are chip markers. | | | | | | | | | | | | | | | | |

**Fig. S1.** Clustering of kinship data in Han Chinese samples.


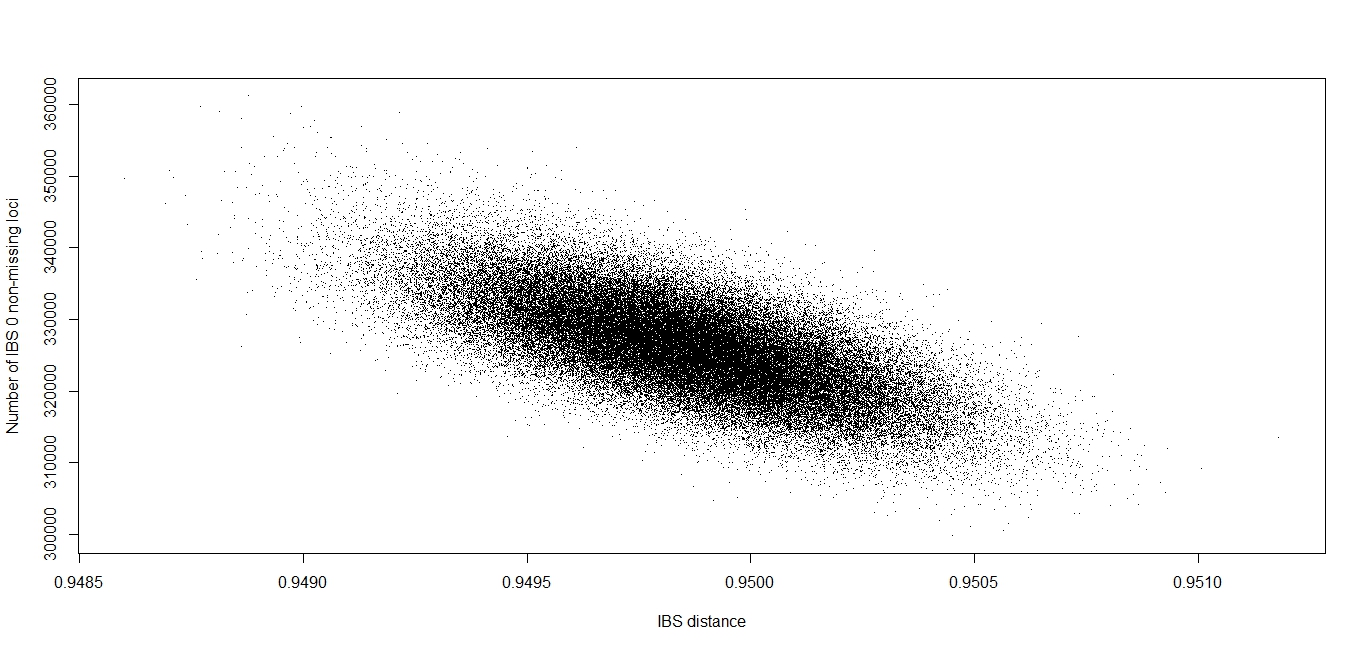


**Fig. S2.** Clustering of population stratification data in Han Chinese samples.


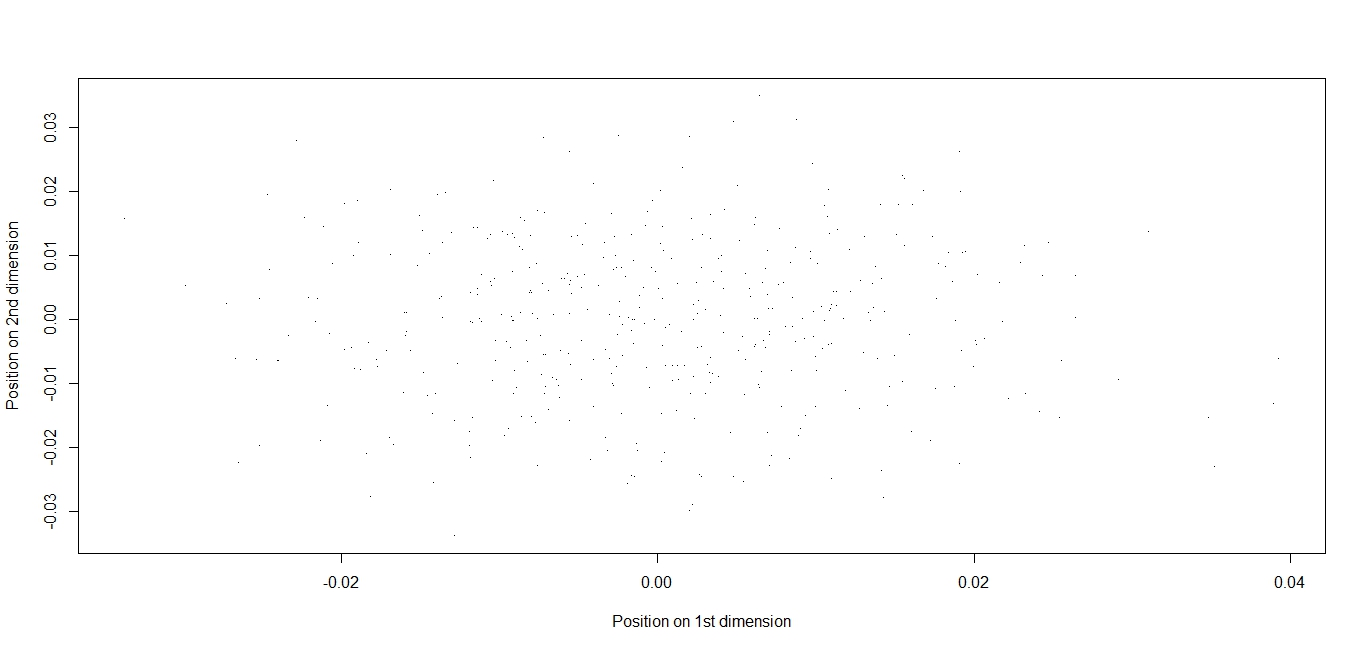

Supplement: Supplementary file 1 [file datasheet1.doc]
